# Feasibility, safety and patient perceptions of exercise-based cardiac telerehabilitation in a multicentre real-world setting after myocardial infarction—the remote exercise SWEDEHEART study

**DOI:** 10.1093/ehjdh/ztaf014

**Published:** 2025-03-04

**Authors:** Maria Bäck, Margret Leosdottir, Mattias Ekström, Kristina Hambraeus, Annica Ravn-Fischer, Sabina Borg, Madeleine Brosved, Marcus Flink, Kajsa Hedin, Charlotta Lans, Jessica Olovsson, Charlotte Urell, Birgitta Öberg, Stefan James

**Affiliations:** Department of Occupational Therapy and Physiotherapy, Sahlgrenska University Hospital, Vita Stråket 13, 413 45 Gothenburg, Sweden; Department of Molecular and Clinical Medicine, Institute of Medicine, Sahlgrenska Academy, University of Gothenburg, Bruna Stråket 16, 413 45 Gothenburg, Sweden; Division of Physiotherapy, Department of Health, Medicine and Caring Sciences, Linköping University, 581 83 Linköping, Sweden; Department of Cardiology, Skåne University Hospital, Malmö, Sweden; Department of Clinical Sciences Malmö, Lund University, Malmö, Sweden; Division of Cardiovascular Medicine, Department of Clinical Sciences, Danderyd Hospital, Stockholm, Sweden; Department of Cardiology, Falu Hospital, Falun, Sweden; Department of Molecular and Clinical Medicine, Institute of Medicine, Sahlgrenska Academy, University of Gothenburg, Bruna Stråket 16, 413 45 Gothenburg, Sweden; Division of Physiotherapy, Department of Health, Medicine and Caring Sciences, Linköping University, 581 83 Linköping, Sweden; Department of Occupational Therapy and Physiotherapy, Sahlgrenska University Hospital, Vita Stråket 13, 413 45 Gothenburg, Sweden; Department of Occupational Therapy and Physiotherapy, Östersund Hospital, Östersund, Sweden; Department of Physiotherapy, Sundsvall Hospital, Sundsvall, Sweden; Department of Physiotherapy, Region Kalmar County, Kalmar County Hospital, Kalmar, Sweden; Department of Health, Medicine and Caring Sciences, Linköping University, Linköping, Sweden; Department of Health and Caring Sciences, Linnaeus University, Växjö, Sweden; Department of Physiotherapy, Region Kronoberg County, Ljungby, Sweden; Department of Women’s and Children’s Health, Uppsala university, Uppsala, Sweden; Division of Physiotherapy, Department of Health, Medicine and Caring Sciences, Linköping University, 581 83 Linköping, Sweden; Uppsala Clinical Research Center, Uppsala University, Uppsala, Sweden; Department of Medical Sciences, Uppsala University, Uppsala, Sweden

**Keywords:** Cardiac rehabilitation, Coronary artery disease, Exercise, Secondary prevention, eHealth, Telerehabilitation

## Abstract

**Aims:**

Cardiac telerehabilitation addresses common barriers for attendance at exercise-based cardiac rehabilitation (EBCR). Pragmatic real-world studies are however lacking, limiting generalizability of available evidence. We aimed to evaluate feasibility, safety, and patient perceptions of remotely delivered EBCR in a multicentre clinical practice setting after myocardial infarction (MI).

**Methods and results:**

This study included 232 post-MI patients (63.7 years, 77.5% men) from 23 cardiac rehabilitation centres in Sweden (2020–22). Exercise was delivered twice per week for 3 months through a real-time group-based video meeting connecting a physiotherapist to patients exercising at home. Outcomes were assessed before and after remote EBCR completion and comprised assessment of physical fitness, self-reported physical activity and exercise, physical capacity, kinesiophobia, health-related quality of life (HRQoL), self-efficacy for exercise, exercise adherence, patient acceptance. Safety monitoring in terms of adverse events (AE) and serious adverse events (SAE) was recorded. A total of 67.2% of the patients attended ≥ 75% of prescribed exercise sessions. Significant improvements in physical fitness, self-reported exercise, physical capacity, kinesiophobia, and HRQoL were observed. Patients agreed that remote EBCR improved health care access (83%), was easy to use (94%) and found exercise performance and interaction acceptable (95%). Sixteen exercise-related AEs (most commonly dizziness and musculoskeletal symptoms) were registered, all of which were resolved. Two SAEs requiring hospitalization were reported, both unrelated to exercise.

**Conclusion:**

This multicentre study supports remote EBCR post-MI as feasible and safe with a high patient acceptance in a real-world setting. The clinical effectiveness needs to be confirmed in a randomized controlled trial.

**Trial registration number:**

NCT04260958.

## Graphical Abstract

**Graphical Abstract:**
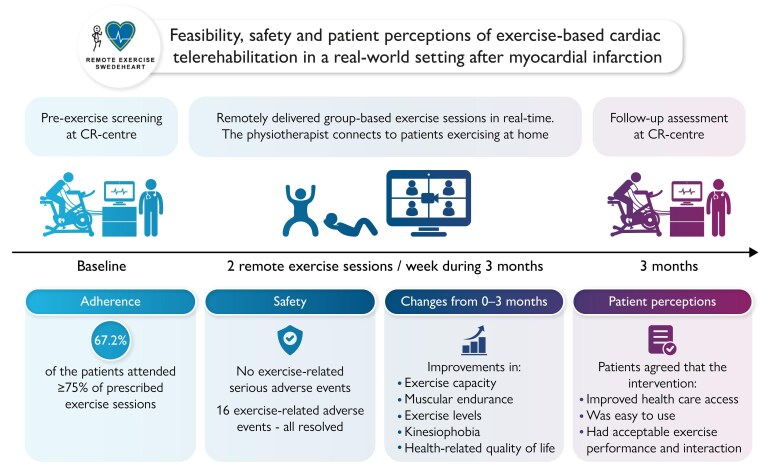

## Introduction

While mortality after an acute myocardial infarction (MI) has declined,^1^ patients are at high risk for recurrent MI,^2^ partially due to insufficient implementation of secondary prevention measures, including comprehensive cardiac rehabilitation (CR) programmes.^3^

Core components of a multidisciplinary CR programme are well defined and should include individual patient assessment, management and control of cardiovascular risk factors, physical activity counselling, prescription of exercise training, dietary advice, psychosocial management, vocational support, and lifestyle behaviour change including patients’ adherence and self-management.^4^ The intervention in the present study specifically targeted exercise as part of CR, often referred to as exercise-based cardiac rehabilitation (EBCR). EBCR has been identified as a key factor in CR as it has been shown to reduce cardiovascular mortality by 42%, hospital readmission by 42% and risk of recurrent MI by 28%.^5^ Additionally, participation in centre-based EBCR is associated with improved aerobic capacity and health-related quality of life (HRQoL)^5,6^ and reduced levels of kinesiophobia (fear of movement).^7^ Participation in EBCR has therefore been given a class 1A recommendation in international guidelines and should be offered to all patients after MI.^8,9^

Despite the clear benefits, participation in centre-based EBCR remains sub-optimal,^3,10^ which limits the effects of the treatment. Main barriers for non-participation include clinical factors, health system factors and logistical factors, such as long travel times, lack of transport and living in geographically inaccessible areas.^11^ Cardiac telerehabilitation provides an opportunity to overcome barriers to attendance by combining the accessibility of home-based EBCR with specialist monitoring, interaction, and support inherent to centre-based EBCR.^12,13^ During the past decade, initial evidence has suggested that remotely delivered EBCR may be an effective alternative to centre-based EBCR in improving health outcomes, such as aerobic capacity, physical activity levels and HRQoL in patients with coronary artery disease (CAD).^14^ Current studies are however limited to single-centre randomized controlled trials with a small sample size^12,14,15^ and there is a lack of studies from real-world settings including more unselective consecutive enrolment. To facilitate implementation into clinical practice, knowledge of patients’ perceptions of pragmatic cardiac telerehabilitation interventions is important.^16^ Therefore, the aim of this study was to evaluate feasibility, safety, changes in physical fitness, and patient-reported outcome measures (PROMs), including self-reported health-related quality of life, kinesiophobia, self-efficacy, level of physical activity, exercise, physical capacity, and patient perceptions of cardiac telerehabilitation delivered as remote EBCR, in a multicentre real-world setting post-MI.

## Methods

### Design

The Remote Exercise SWEDEHEART study is a national multicentre study performed in two parts: (i) a feasibility and safety study (cohort design) and (ii) a registry-based cluster-randomized crossover clinical trial.^17^ This manuscript reports the results from part 1. Feasibility in this manuscript is defined as the acceptability and demand of the intervention to guide whether it can be delivered to intended patients regarding inclusion, implementation, practicality, and limited efficacy testing. There are no defined cut-offs as such, but preliminary positive results can suggest that an intervention is ready to be tested in a full-scale trial.

SWEDEHEART (the Swedish Web-system for Enhancement and Development of Evidence-based care in Heart disease Evaluated According to Recommended Therapies) is a national quality registry that continuously records the quality and content of patient care, treatments, and outcomes after MI.^18^ The sub-registry auditing information on secondary prevention and CR performance after MI is referred to as the SWEDEHEART-CR registry (nationally known as SEPHIA).^19^ SWEDEHEART-CR has a 100% coverage at centre-level with a 75–80% uptake of all patients <80 years of age with an MI. More than 80 variables describing CR performance are registered at time-fixed follow-up visits, including two visits to a nurse or physician, at 2 months and 1-year after MI, and two visits to a physiotherapist at start and end of an exercise-based CR programme. Patients in the present study were offered to participate in a comprehensive CR programme as part of usual care; however, only the exercise intervention was delivered remotely and was as such specifically evaluated in the present study.

### Study population

The study inclusion criteria were a diagnosis of type 1 MI (ICD codes I21 or I22) included in the SWEDEHEART-CR registry and age 18–79 years at discharge from hospital. Exclusion criteria were incomplete coronary revascularization defined as at least one remaining hemodynamically significant stenosis, severe valve or structural heart disease, heart failure with New York Heart Association class III–IV, serious arrhythmias, inability to understand or speak Swedish, no internet access at home, pathological exercise test according to standard criteria^20^ indicating high risk for adverse events (AEs) during remote EBCR, more than 6 months between discharge from hospital and screening or any other condition that might interfere with the ability of the patient to comply with the study protocol.

### Procedure

A total of 23 CR centres with wide geographical distribution across the country participated in the study. Screening started at two pilot centres in February 2020 and most of the other centres entered the study in Q1-Q2, 2021. The last patient screened was in May 2022. Check of eligibility and inclusion of patients followed clinical routine at each study centre and was commonly performed during the index MI hospitalization or at the first CR physiotherapy visit. As most parts of the study were conducted during the COVID-19 pandemic, study centres needed to adapt their study participation according to the current clinical situation and available personnel. Normally, only the remote EBCR was offered to patients. At some CR centres, centre-based EBCR was also partly available during the study period depending on the COVID-19 pandemic.

Baseline data, as well as most outcome data were collected as part of usual care and was retrieved from the SWEDEHEART registry.^18,19^ Data from the added study-specific questionnaires were manually entered into an electronic case report form (eCRF) by physiotherapists at each study centre. Participation in remote- and home-based exercise sessions, registrations of AE and serious adverse events (SAE) were also entered in the eCRF.

The study was approved by the Swedish Ethical Review Authority (registration number 2019–03958, with amendments 2020-00514, 2021-03951, 2022-03197-02, and 2022-03547-02) and was carried out in accordance with Good Clinical Practice and the Declaration of Helsinki. The study is registered at ClinicalTrials.gov (Identifier: NCT04260958). An informed consent was retrieved before patients performed any study-specific interventions.

### Intervention

Patients attended one physiotherapy visit according to usual care visits at each hospital, ∼2–4 weeks after discharge, prior to starting the remote EBCR programme to perform a pre-exercise screening, including tests of physical fitness. Cardiologists were responsible for medical safety. The remote EBCR programme was delivered through a real-time group-based video meeting, by connecting a physiotherapist at the CR centre to patients exercising at home or another self-selected place. The groups consisted of in average 6–10 patients, depending on number of included patients at each centre. Peers could see and interact with each other and with the physiotherapist during the EBCR sessions. CR centres were encouraged to use teleconferencing systems already implemented at their hospital. Routines in case of emergency were established. When the remote EBCR session started, the physiotherapist asked each patient, which current address they were on. In case of a SAE, the physiotherapist could immediately alert an ambulance to the address, while other personnel at the CR centre took care of the reactions of the remaining patients. In case of a sub-acute event, such as dizziness or pain, the physiotherapist handled this remotely and contacted the medical responsible cardiologist for a further medical assessment, if relevant.

The remote EBCR programme was delivered according to international standards, including 60 min of aerobic and muscle strength exercises, twice a week for 3 months (24 sessions). Attending ≥ 75% of the 24 sessions over a 4-months period was considered as successful participation.^19^ Each remote EBCR session started with a warm-up period followed by aerobic exercises performed in 45 s intervals with a high intensity level (Borg rating of perceived exertion scale, RPE 14–17) with 15 s periods at moderate intensity (Borg RPE 12–13) between intervals for a total duration of 25 min. The muscle strength training consisted of 11 exercises with an elastic band and on the floor, including various muscle groups and was performed in 12 repetitions × 2 sets. The remote EBCR programme ended with a cool-down period. The included exercises were individually tailored based on pre-exercise screening tests and progressed during the exercise period. Before and after each session, patients were offered an individual conversation with the physiotherapist. In average, patients took the opportunity of individual conversation once every other week.

In addition, patients were asked to perform one additional home-based session of at least 30 min aerobic exercise per week at an intensity corresponding to Borg RPE 13–15 and to register these sessions in an exercise diary. Diary registrations were followed-up by physiotherapists at the remote exercise sessions. After having completed the remote EBCR programme, follow-up tests of physical fitness with the physiotherapist were conducted at the CR centre.

### Study variables

The SWEDEHEART registry was used for collecting baseline demographic data. To assess changes in physical fitness and PROMs at start and end of EBCR (3–4 months after EBCR start), the physiotherapy variables included in the SWEDEHEART registry and implemented in clinical practice were used. A set of specific study questionnaires were also administered.

#### Variables in SWEDEHEART

Tests of physical fitness:

- Exercise capacity on a symptom-limited bicycle ergometer test with an increased workload of 25 W every 4.5 min. The exercise test is discontinued at Borg RPE 17 and/or dyspnoea 7 at Borg’s CR-10 scale.^21^ Standard criteria for discontinuation were used, such as increasing chest pain and drop in blood pressure.^20^ Maximal power was calculated according to the Strandell formula.^22^
- Muscular endurance tests include a unilateral isotonic shoulder flexion (maximum number of repetitions) and a unilateral isotonic heel lift (maximum number of repetitions). The tests are described in detail elsewhere.^21^

Patient-reported outcomes measures:

- Two questions on the frequency of physical activity (moderate-intensity physical activity accumulated towards the 30-min minimum by performing bouts each lasting 10 or more minutes) and exercise (vigorous-intensity physical activity/exercise for a minimum of 20 min) during the past week according to Haskell. Patients are asked to mark the number of days (0–7) that corresponds to the indicated activity level for each question.^23^
- Physical activity level during the past week according to Frändin and Grimby questionnaire [Min:1 = hardly any physical activity, Max:6 = Hard or very hard exercise regularly and several times a week, where the physical exertion is great.^24^
- Two questions on physical capacity (i) visual analogue scale (VAS) where patients rate their current physical capacity according to 100 = best possible physical capacity, 0 = worst possible physical capacity. (ii) A dichotomous question: Do you experience any limitation in everyday life due to your current physical capacity? (yes or no). If yes, patients chose the most relevant reason, i.e. chest pain, fatigue, fear of movement, shortness of breath.

#### Study-specific variables

- HRQoL, evaluated with the EQ-VAS. The EQ-VAS records the patient’s self-rated health on a vertical analogue scale where the endpoints are labelled ‘the best health you can imagine (=100) and the worst health you can imagine’ (=0).^25^
- Kinesiophobia (fear of movement), as measured by the Tampa Scale for Kinesiophobia Heart (17 items, ordinal scale, 1 = strongly disagree to 4 = strongly agree). A score >37 points is defined as occurrence of kinesiophobia.^26^
- Self-efficacy as measured with the self-efficacy for Exercise Scale (9 items, ordinal scale, 0 = not confident to 10 = very confident in ability to exercise). The scale was scored by summing the numerical ratings for each item, divided by the number of items.^27^
- At baseline, patients were asked to fill in two questions recommended by the National Board of Health and Welfare regarding their previous level of moderate physical activity (ordinal scale 1 = 0 min to 7= > 300 min) and exercise (1 = 0 min to 6=>120 min).^28^ Patients also answered a question on time spent sitting during a usual day (1 = virtually all day to 7 = never).^29^

### Adherence to exercise

Attending ≥75% of the 24 recommended remote EBCR sessions over a 4-month period was considered as being adherent to the remote EBCR programme, while performing ≥75% of the 12 recommended additional home-based exercise sessions at the prescribed intensity and duration was defined as being adherent to home-based exercise.

### Assessment of safety

Safety monitoring in terms of AE and SAE was recorded and on each exercise occasion patients were asked whether they had experienced any AEs that could be related to the exercise. Ongoing AEs were followed-up at the next exercise sessions. In this study, an AE was defined as any unfavourable, unintended clinical sign, symptom, medical complaint or clinically relevant change with at least a possible relationship to the EBCR. An SAE is any untoward medical occurrence or effect that results in death, is life-threatening, requires hospitalization, results in persistent or significant disability or incapacity or other important medical events. All AEs and SAEs were registered in the eCRF. If an event was serious according to the definitions, it was also reported on a separate SAE-form and sent to the sponsor representative. Safety was defined as the absence of SAEs.

### Patient perceptions of remote EBCR

At end of EBCR patients filled in a study-specific questionnaire evaluating patient acceptance of remote EBCR and their perceived degree of usability with responses recorded on a five-point Likert scale (1 = do not agree at all and 5 = completely agree). The questions were inspired by the Telehealth Usability Questionnaire by Parmanto *et al.*^30^ that evaluates usability of telehealth services and implementation in six dimensions. The questions were modified to suit the aim of the present study:

1. *Usefulness* (two questions) refers to the patient’s perception of how the remote EBCR functions to provide a healthcare service similar to the in-person centre-based programme.
2. *Ease of use and learnability* (four questions) contains questions on whether remote EBCR was simple to use and if it was easy to learn how to use.
3. *Interface quality* (one question) refers to whether the system had all the expected functionality and capabilities.
4. *Interaction quality* (11 questions) measures the patient’s perceived interaction with the physiotherapist and peers, including the quality of audio and video and the similarity of the remote interaction to an in-person interaction.
5. *Reliability* (three questions) refers to whether remote EBCR is as reliable as an in-person visit.
6. *Satisfaction and future use* (three questions) are related to overall patient satisfaction and how willing the patient would be to participate in remote EBCR in the future.

### Statistical analysis

Categorical data are presented as absolute and relative frequencies, whereas continuous data are presented as means and standard deviations (SDs) or medians with quartiles (Q1-Q3), as appropriate. Changes from baseline to follow-up were analysed with Wilcoxon signed rank test for continuous data while categorical data was analysed with a χ^2^ test. We used the full dataset without any imputation of missing values, except for the TSV-SV Heart where internal missing was imputed by calculating the mean value for the answers given multiplied by 17. A sensitivity analysis excluding the patients that had performed centre-based or hybrid EBCR was performed. The statistical significance was set at *P* < 0.05. Statistical analysis was performed using SPSS version 28 (IBM, Armonk, New York, USA).

## Results

**Figure 1:**
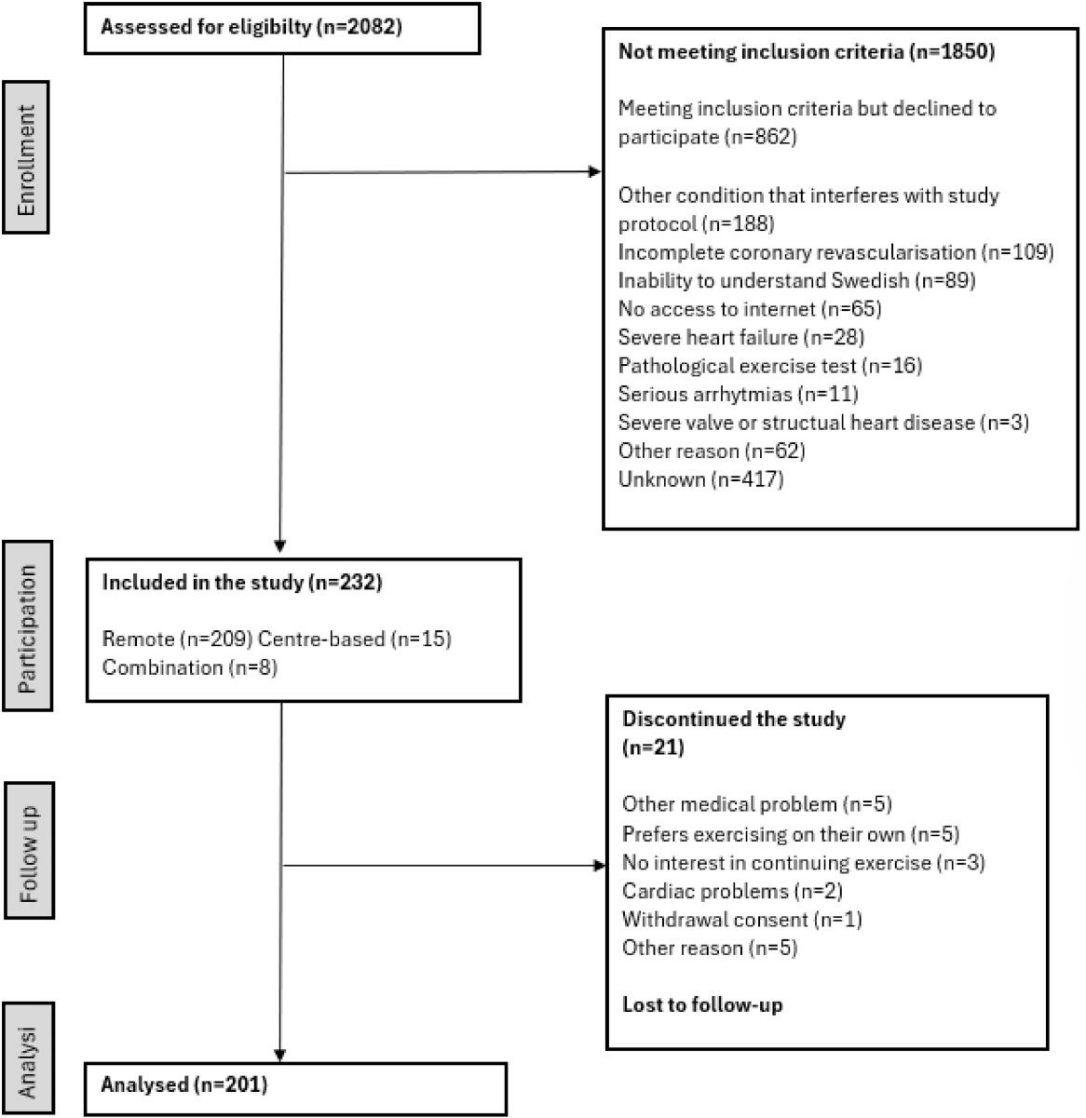
PRISMA flow diagram of the screening process.

Out of 2082 patients who were screened for eligibility, 862 patients met the inclusion criteria but declined to participate. The most commonly reported exclusion criteria were other conditions that interfered with the study protocol (*n* = 188), incomplete coronary revascularization (*n* = 109), and inability to understand Swedish (*n* = 89). Out of the 232 patients that started the intervention, data from *n* = 201 were analysed. Due to various reasons, mostly patient willingness and safety reasons, 15 out of the 201 patients switched to centre-based exercise and eight patients performed hybrid EBCR, with a combination of centre-based and remotely delivered exercise. The study flow chart is shown in *Figure 1*. A detailed presentation of screened and included patients per hospital is given as supplementary material online, *Table S1*.

Patient characteristics at baseline are shown in *Table 1*. The mean age was 63.7 ± 8.9 years, and *n* = 169 (77.5%) were men. A total of *n* = 100 (46.7%) of the patients had a diagnosis of ST-elevation MI, *n* = 192 (88.9%) had undergone a percutaneous coronary intervention, *n* = 143 (71.9%) had a normal (>50%) left ventricular ejection fraction and *n* = 128 (55.9%) of the patients met the general physical activity recommendations.

**Table 1:** Baseline patient characteristics (N = 232)

Variable	Internal missing, *n* (%)	Mean (SD) or number (%)
Sex, men, *n* (%)	14 (6.0)	169 (77.5)
Age, mean ± SD	14 (6.0)	63.7 ± 8.9
BMI, mean ± SD	28 (12.1)	27.5 ± 4.4
Marital status, *n* (%)	2 (0.9)	
Married		194 (83.6)
No partner/divorced/widow/widower		36 (15.5)
Occupation status, *n* (%)	49 (21.1)	
Employed		75 (41.0)
On sick leave		16 (8.7)
Retired		88 (48.1)
Other		4 (2.2)
Smoking status, *n* (%)	49 (21.1)	
Never smoker		99 (54.1)
Former smoker > 1 month		78 (42.6)
Current smoker		5 (2.2)
Previous physical activity level		
Sedentary time, hours/day	9 (3.9)	5 (5–8)
Exercise, min/week	3 (1.1)	45 (30–105)
Daily physical activity, min/week	2 (0.9)	225 (75–225)
Meeting physical activity recommendations, *n* (%)	3 (1.1)	128 (55.9)
Previous disease, *n* (%)		
Previous ischaemic heart disease	14 (6)	10 (4.6)
Previous diabetes, *n* (%)	31 (13.4)	16 (7.5)
Previous hypertension, *n* (%)	14 (6)	103 (47.2)
Type of MI, *n* (%)	18 (7.8)	
STEMI		100 (46.7)
Non-STEMI		111 (51.9)
Current PCI, *n* (%)	16 (6.9)	192 (88.9)
Current CABG, *n* (%)	21 (9.5)	14 (6.6)
Medication at discharge, *n* (%)		
ACE-inhibitors or ARB	15 (6.5)	185 (85.3)
SGLT-2 inhibitors	31 (13.4)	13 (6.5)
Betablocker	14 (6.0)	169 (77.5)
Aldosterone antagonists	20 (8.6)	4 (1.9)
Diuretics	14 (6.0)	16 (7.3)
Lipid lowering therapy	25 (10.8)	204 (98.6)
Dual antiplatelet therapy	27 (11.6)	201 (98.0)
Left ventricular function, *n* (%)	33 (14.2)	
Normal (>50%)		143 (71.9)
Mildly reduced (40–49%)		44 (22.1)
Moderately reduced (30–39%)		12 (6.0)
Participation in a patient educational programme after discharge	49 (21.1)	36 (15.5)

Previous ischaemic heart disease, myocardial infarction, percutaneous coronary intervention, coronary artery bypass grafting; meeting physical activity recommendations were calculated by multiplying time in exercise by two plus time in physical activity. Dual antiplatelet therapy: aspirin + P2Y12 inhibitor or single antiplatelet therapy + oral anticoagulant; lipid lowering therapy: statin, ezetimibe, and/or PCSK9i.

BMI, body mass index; STEMI, ST-elevation myocardial infarction; non-STEMI, non-ST-elevation myocardial infarction; PCI, percutaneous coronary intervention; CABG, coronary artery bypass grafting; ACE, angiotensin converter enzyme; ARB, angiotensin II receptor blocker 2; SGLT-2, sodium–glucose cotransporter-2.

### Adherence to exercise

Patients performed in median 20 (Q1 15 – Q3 24) remote EBCR sessions and 11 (Q1 6 – Q3 14) home-based exercise sessions. A total of *n* = 133/198 (67.2%) and *n* = 92/152 (60.5%) of the patients were adherent to the remote EBCR programme and home-based exercise programme, respectively. When assessing the combined adherence from remote EBCR and home-based exercise, *n* = 76/154 (49.4%) of patients were found to be adherent. Reasons for non-adherence to the remote EBCR programme are given in *Table 2*.

**Table 2:** Exercise adherence

Adherence		
Documented reasons for not participating in EBCR	Participation 0 sessions (*n* = 11)	Participation 1–17 sessions (*n* = 57)
Cancelled session	3	24
Not interested in supervised exercise	0	3
Medical problem	2	3
EBCR was not available	0	5
Lack of time	1	6
Combination of several of the documented reasons	0	4
Unknown	4	12
Missing	1	0

### Adverse and serious adverse events

A total of 16 AEs were registered during exercise, including dizziness (*n* = 4), fatigue (*n* = 3), musculoskeletal symptoms (=3), nausea (*n* = 2), chest pain (*n* = 1), syncope (*n* = 1), fall in blood pressure (*n* = 1), and dyspnoea (*n* = 1). In the more serious cases, patients needed to terminate the remote EBCR session and physiotherapists contacted the medically responsible cardiologist for further assessment. All AEs were resolved and did not cause any sequelae. Two SAEs that required hospitalization were reported during the period the patients were included in the study (chest pain and in-stent restenosis), but both were unrelated to exercise.

### Changes from baseline to follow-up

Assessments of changes from baseline to 4-month follow-up demonstrated statistically significant improvements in aerobic physical capacity (*P* < 0.001), muscular endurance (*P* < 0.001), self-reported level of exercise, physical activity, and physical capacity (*P* < 0.001) and HRQoL (*P* < 0.001) (*Table 3*). A smaller proportion reported limitations in everyday activities due to current physical capacity (*P* < 0.001) and fewer patients reported kinesiophobia (*P* < 0.001). The sensitivity analysis showed no statistically significant differences when excluding patients that had performed centre-based or hybrid EBCR.

**Table 3:** Changes in physical fitness and patient-reported outcome measures between baseline and follow-up

Variable	Number of patients	Baseline	4 months follow-up	*P*-value
Variables in SWEDEHEART				
Physical capacity				
Aerobic capacity, watts	164	100 (75–125)	111 (92–138)	<0.001
Shoulder flexion, maximum number of	138	30 (22–40)	37 (28–49)	<0.001
Heel lift, maximum number of	138	17 (11–24)	22 (15–30)	<0.001
Self-reported level of physical activity and exercise				
Haskell questionnaire, number of days				
Question 1, Physical activity	156	5 (3–7)	5 (3–7)	0.09
Question 2, Exercise	156	1 (0–3)	2 (2–3)	<0.001
Frändin and Grimby questionnaire, Level	156	3 (3–4)	4 (4–5)	<0.001
Self-reported physical capacity				
Self-rated physical capacity, visual analogue scale	152	50 (31–65)	73 (62–80)	<0.001
Limitation in everyday life due to current physical capacity? *n* (%)	156	Yes: 70 (45)	Yes: 38 (24)	<0.001
		No: 86 (55)	No: 118 (76)	
Study-specific variables				
Tampa Scale for Kinesiophobia Heart, sum score	191	27 (24–33)	25 (22–29)	<0.001
Tampa Scale for Kinesiophobia Heart >37 points, *n* (%)	191	20 (10)	10 (5)	<0.001
EQ5D-VAS	194	70 (50–80)	80 (70–90)	<0.001
Self-efficacy for Exercise Scale	178	62 (37–76)	60 (44–76)	0.389

Interval data are presented as median (25th-75th percentile). Nomimal data are presented as number (%). Interval data were analysed with Wilcoxon test and nominal data with χ^2^ test.

### Patient perceptions of remote EBCR

Patients agreed that remote EBCR improved access to health care, *n* = 142/172 (83%) and saved time traveling to a centre-based facility, *n* = 130/172 (76%). Almost all patients agreed that it was easy to learn, *n* = 163/172 (95%) and use 159/170 (94%) the remote EBCR videoconferencing system and that the system had the desirable functionalities, *n* = 151/171 (88%). Regarding exercise performance, ∼95% of the patients found it acceptable to perform both aerobic exercises, *n* = 165/173, exercises with elastic bands, *n* = 167/172, and strength training on the floor, *n* = 160/173. In terms of the interaction quality items, *n* = 163/172 (95%) of the patients perceived that they could call on attention if needed and that they could easily talk with, *n* = 159/173 (92%), see *n* = 165/171 (96%) and hear *n* = 163/172 (95%) the physiotherapist. Additionally, *n* = 100/164 (61%) of the patients perceived that they could easily talk to, see *n* = 127/169 (75%) and hear *n* = 128/169 (76%) their peers. On the other hand, *n* = 16/169 (10%) and *n* = 19/169 (11%), respectively, disagreed or partially disagreed that they could see and hear their peers. Additionally, *n* = 31/164 (19%) disagreed or partially disagreed that they could satisfactorily talk with the peers.

**Figure 2:**
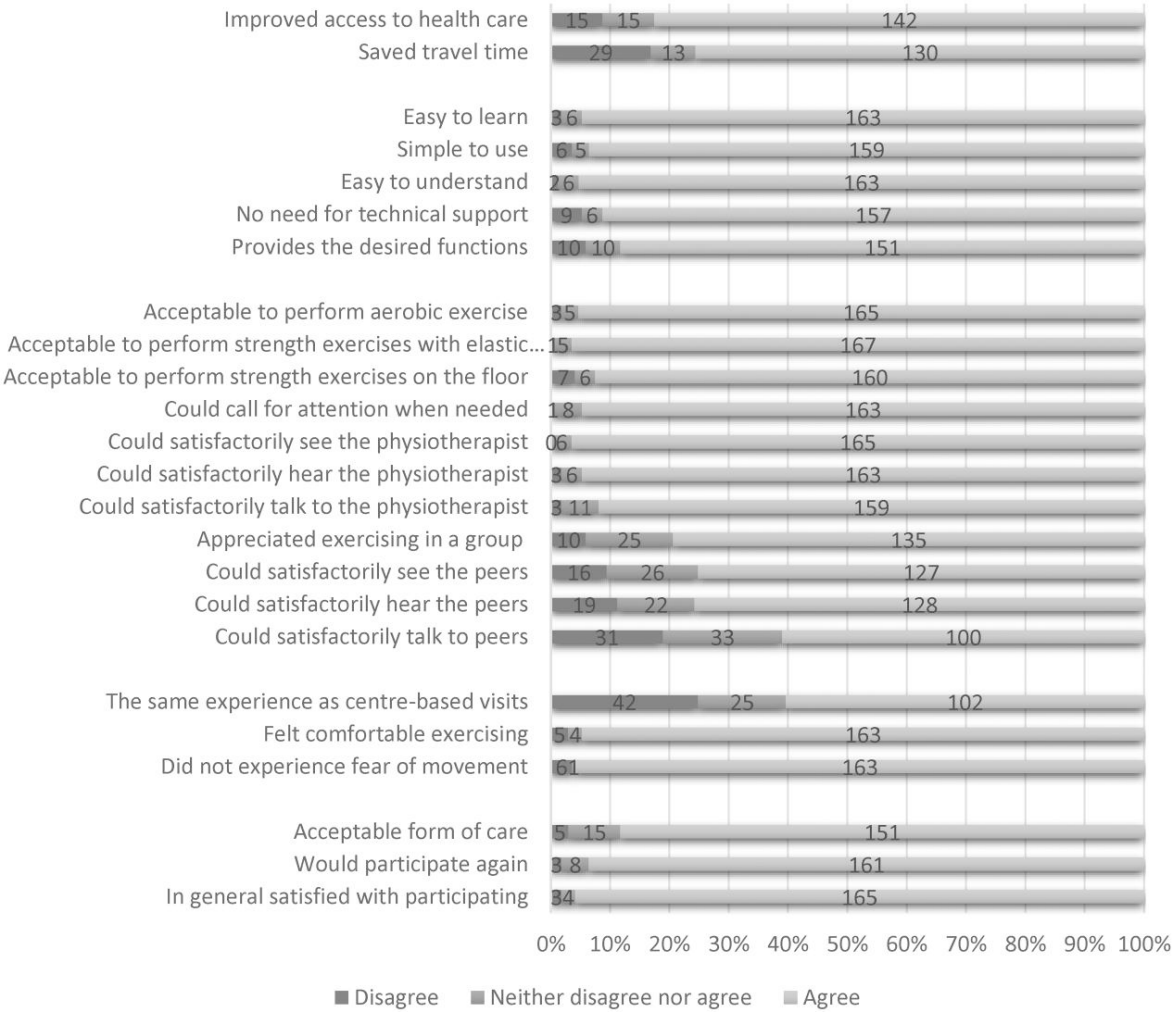
Patient perceptions of remote exercise-based cardiac rehabilitation.

Sixty percent, *n* = 102/169, of the patients found the remote interaction equal to an in-person interaction, while *n* = 42/169 (25%) disagreed or partially disagreed to this statement. A total of *n* = 151/171 (88%) of the patients found that remote EBCR was an acceptable form of delivering health care, *n* = 165/172 (96%) of the patients agreed that they were overall satisfied with the remote EBCR programme and *n* = 161/172 (94%) were willing to participate again in the future (*Figure 2*). A more detailed presentation of the items is found in *Table 4*.

**Table 4:** Patient perceptions of remote exercise-based cardiac rehabilitation, questionnaire item statistics

Item	Number	Median (Q1-Q3)	Skewness	Kurtosis
Remote EBCR improves my access to healthcare services	172	5 (4–5)	−1.7	2.2
For me it was essential to save time by participating in remote EBCR	172	5 (4–5)	−1.2	0.1
It was easy to learn to use the digital system	172	5 (5–5)	−3.2	12.1
It was easy to use the digital system	170	5 (5–5)	−3.0	9.5
The digital system was unnecessarily difficult	171	1 (1–1)	4.0	16.2
It would have been desirable to get technical support	172	1 (1–1)	2.8	7.5
The digital system provides the desired functions	171	5 (4–5)	−2.4	5.1
It was acceptable to perform aerobic exercises	173	5 (5–5)	−3.3	12.5
It was acceptable to perform strength exercises with an elastic band	173	5 (5–5)	−3.4	15.6
It was acceptable to perform strength exercises on the floor	173	5 (5–5)	−2.8	8.0
I could call for attention whenever needed	172	5 (5–5)	−3.0	11.3
I could satisfactorily see the physiotherapist using the digital system	171	5 (5–5)	−2.5	5.6
I could satisfactorily hear the physiotherapist using the digital system	172	5 (5–5)	−2.8	9.5
I could satisfactorily talk with the physiotherapist using the digital system	173	5 (5–5)	−2.2	4.1
I appreciated exercising in a group using the digital system	170	5 (4–5)	−1.4	1.4
I could satisfactorily see the peers using the digital system	169	5 (3.5–5)	−1.3	0.7
I could satisfactorily hear the peers using the digital system	169	5 (4–5)	−1.3	0.7
I could satisfactorily talk with the physiotherapist using the digital system	164	4 (3–5)	−0.7	−0.7
I think the visits provided over the digital system are the same as in-person visits	169	4 (2.5–5)	−0.6	−0.9
I felt comfortable exercising using the digital system	172	5 (5–5)	−3,5	13.4
I experienced fear of movement when exercising using the digital system	170	1 (1–1)	4.7	21.7
Healthcare visits using a digital system is an acceptable form of care	171	4 (5–5)	−2.0	4.4
I would accept participating in remote EBCR again	172	5 (5–5)	−2.9	9.2
Overall, I am satisfied with participating using remote EBCR	172	5 (5–5)	−3.7	16.5

EBCR, exercise-based cardiac rehabilitation.

## Discussion

Cardiac telerehabilitation has the potential to address many of the challenges with traditional centre-based EBCR in terms of low participation rates. Results from previous cardiac telerehabilitation interventions are promising, but studies have often been small randomized controlled trials limited to research settings and results are rarely implemented in regular care.^14,31^ The current multicentre study adds to existing literature by showing that remote EBCR delivered in real-time is feasible and safe with a high patient acceptance in a setting of patients post-MI, representative of everyday clinical practice.

To reach the potential benefits of cardiac telerehabilitation, the delivery system must be usable for patients. Patients in the present study agreed to a high extent that the remote EBCR system was easy to learn and use, that exercising worked out well in a home environment and that they could easily interact with the physiotherapist. Most patients agreed that they could easily interact also with peers; however, around 10% of the patients disagreed or partially disagreed. Results from a qualitative study from our group showed that some patients found exercising with peers as an important part of a remotely delivered EBCR programme, while others expressed that they primarily wanted to focus on their own performance and did not took big notice about the other patients.^32^ A systematic scoping review on acceptance of home-based comprehensive cardiac telerehabilitation programmes confirmed that patients valued individualized exercise prescription, remote supervision and the ability to communicate with health care professionals.^16^ Moreover, most patients in the present study agreed that they were satisfied with the remote EBCR programme and were willing to participate again in the future. These are important findings since recognizing patient acceptance and usability of cardiac telerehabilitation programmes is crucial for successful implementation into clinical practice.^13^ However, as 25% of the patients in our study disagreed or partially disagreed that the experience of a digitally delivered meeting with a health care professional was equally good as an in-person meeting, offering both alternatives and hybrid solutions are suggested for the future.

The relative improvement of 11% in aerobic capacity from baseline to follow-up in our study is similar to data from the SWEDEHEART registry (11.2% improvement), in a comparable group of real-world patients after MI attending usual care EBCR.^10^ Additionally, the relative change in self-reported levels of physical activity, exercise and physical capacity were similar to registry-based data.^10^ It is worth noting that patients in the present study had a slightly higher aerobic capacity, muscle endurance, and level of self-reported exercise at baseline compared with SWEDEHEART data.^10^ It may be speculated that a healthier group of patients preferred participation in remote EBCR or that less fit patients avoided attending CR at all during the COVID-19 pandemic, when our study was conducted. Our results are in line with results from a recent umbrella review demonstrating that improvements in aerobic capacity and physical activity levels after cardiac telerehabilitation were not significantly different from centre-based CR in patients with CAD.^14^ The included studies were, however, limited by a high level of heterogeneity in patient selection, outcomes and the way cardiac telerehabilitation was delivered, which limits clinical implications in a broader context.^14^

Muscle strength training is recommended as part of an EBCR programme,^4^ strongly supported by results from a meta-analysis showing that combined aerobic and muscle strength training has greater effects on cardiorespiratory fitness and body composition than aerobic training alone in patients with CAD.^33^ Despite clear effects, there is still limited evidence regarding the effects of cardiac telerehabilitation on muscle strength and endurance.^34^ The present study showed that the maximum number of shoulder flexions and heel lifts improved from baseline to follow-up and the size of the relative increase was similar to prior SWEDEHEART data.^10^ In addition, more than 90% of the patients agreed that performing muscle strength exercises with elastic bands and on the floor was an acceptable form of exercise in a cardiac telerehabilitation programme. As such, this modality seems feasible and could be considered for future studies in the field of cardiac telerehabilitation.

It is previously known that high levels of kinesiophobia in patients with CAD are associated with a negative impact on rehabilitation outcomes.^35^ In the present study, 20% of the patients reported a high level of kinesiophobia at the start of remote EBCR, which was reduced to 10% at follow-up. This finding can be compared with a study by Hoeve *et al.*^36^ showing that 40% of a mixed population of patients with heart disease demonstrated high levels of kinesiophobia at the start of centre-based CR, which decreased to 26% post-CR. Even though it may be hypothesized that a larger proportion of patients with kinesiophobia prefer centre-based EBCR, it is promising that kinesiophobia can also be reduced from participation in remote EBCR. A previous study showed that supervised remote EBCR delivered in real-time by trained physiotherapists provides a sense of security for patients, which is important to consider when implementing cardiac telerehabilitation in clinical practice.^32^

In the present study, almost 70% of the patients were adherent to the remote EBCR programme. As exercise sessions were performed in real-time, both participation and reasons for non-participation could be documented with high validity. Commonly reported reasons for non-participation were lack of time, medical problems, and patients cancelling exercise sessions for unknown reasons. As exercise adherence has not been frequently reported in previous cardiac telerehabilitation studies, it is difficult to compare our results. A systematic review on telerehabilitation in patients with cardiopulmonary diseases demonstrated that in the few available studies adherence was high in general but decreased over time and registrations were biased by the use of self-reports such as diaries.^34^ Therefore, factors of importance for adherence to exercise-based cardiac telerehabilitation need to be further explored in future studies.

Our findings suggest that remotely delivered EBCR in real-time is a safe treatment for the majority of real-world patients after MI < 80 years of age representative of clinical practice, with only two reported SAEs requiring hospitalization, which were both unrelated to exercise. For safety reasons, we chose to exclude patients with higher risk for AEs during exercise, for example patients with severe heart failure or malign arrhythmias. Our result can be compared with a previous meta-analysis showing no AEs directly linked to cardiac telerehabilitation, such as mortality or readmission rates.^37^ Results are not directly comparable between studies, however, due to differing patient selection and choice of telerehabilitation models. Additionally, 16 AEs were registered in our study, where the most serious cases required actions from the physiotherapist, which supports synchronous monitoring with real-time interaction between patients and the physiotherapist during remote EBCR. Patient assessment and pre-exercise screening are important parts of EBCR to form the basis for individualized and safe exercise prescription.^8^ While this recommendation is well implemented as part of centre-based CR, it needs to be stressed as equally important in a cardiac telerehabilitation programme. Moving forward, it is crucial for future research to continue investigating and monitoring safety outcomes to further enhance our understanding of potential risks associated with cardiac telerehabilitation.

This study has some strengths and limitations that need to be acknowledged. First, a strength of the study is the inclusion at numerous Swedish CR centres following usual care clinical routines, which minimises the risk of selection bias. This broad inclusion in turn can facilitate implementation of the study results into clinical practice. However, the use of the SWEDEHEART registry meant only patients <80 years of age with knowledge of the Swedish language could be included, and additionally no variables indicating socioeconomic status is included in the registry, which may be considered a limitation in terms of generalizability. Also, the left ventricular diastolic function was not available. In addition, a quite large number of patients assessed for eligibility declined to participate. Commonly reported reasons were that patients did not want to attend the CR-centre during the COVID-19 pandemic and that they preferred exercising by themselves. It had been preferable performing a maximal exercise test, but to be pragmatic we used the sub-maximal exercise test according to usual care in Sweden. Similarly, we used the questionnaires included in the SWEDEHEART registry, while being aware of the limitations of self-reported as compared with objectively assessed physical activity. There was no questionnaire available that could evaluate digital health technology acceptance in relation to the programme-specific components in the remote EBCR programme. Therefore, we developed a study-specific questionnaire with a general acceptance concept with inspiration from the Telehealth Usability Questionnaire.^30^ Psychometric properties were not evaluated, however, which can be considered a limitation.

In summary, this study shows that a remote EBCR programme with a real-time interaction between the physiotherapist and patients post-MI in a real-world setting is safe with a high exercise adherence and patient acceptance. Physical capacity, self-reported physical activity and exercise, and HRQoL were improved, while self-reported kinesiophobia diminished. The goal of developing cardiac telerehabilitation programmes is not to replace centre-based EBCR, but rather to complement the current standard of care. Comparing different cardiac telerehabilitation models, such as telerehabilitation only, or hybrid models with synchronous or asynchronous monitoring, is important for future studies to determine, which procedures may best meet the need of specific patient populations.

## Conclusions

This national multicentre cohort study supports remote EBCR delivered in real-time as feasible and safe with a high patient acceptance and improvements from baseline to follow-up in physical capacity, self-reported physical activity and exercise, health-related quality of life and kinesiophobia in a real-world population post-MI representative of clinical practice. The clinical effectiveness needs to be confirmed in a randomized controlled trial design.

## Lead author biography


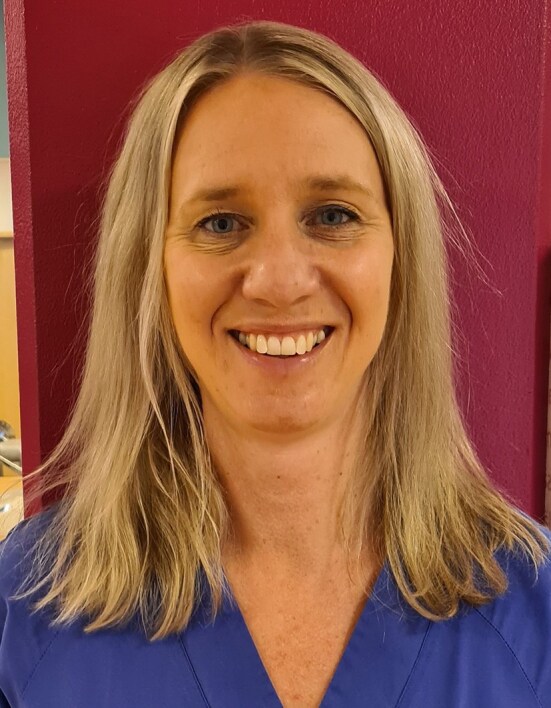


Professor Maria Bäck, RPT, FESC, Institute of Medicine, Sahlgrenska Academy, University of Gothenburg, Sahlgrenska University Hospital and Department of Health, Medicine and Caring Sciences, Linköping University, Sweden. She is board member of the European Association of Cardiovascular Nursing and Allied Professions (ACNAP) and member of the European Association of Preventive Cardiology (EAPC) Secondary Prevention and Rehabilitation Section. Her expertise and research interests cover physical activity and exercise-based cardiac rehabilitation as part of secondary prevention, with a special focus on telerehabilitation.

## Supplementary Material

ztaf014_Supplementary_Data

## Data Availability

The data underlying this article will be shared on reasonable request to the corresponding author.
