# Supplementary material for: Feasibility, safety and patient perceptions of exercise-based cardiac telerehabilitation in a multicentre real-world setting after myocardial infarction—the remote exercise SWEDEHEART study
Source: Eur Heart J Digit Health. 2025 Mar 4;6(3):508–18. doi: 10.1093/ehjdh/ztaf014 (PMC12088728; doi:10.1093/ehjdh/ztaf014)
Supplement: ztaf014_Supplementary_Data [file ztaf014_supplementary_data.docx]

**Cardiac rehabilitation centres screening and inclusion of patients**

| Site | Start of inclusion | End of  inclusion | Screened Patients | Included  patients |
| --- | --- | --- | --- | --- |
| Sahlgrenska | 20-02-11 | 22-03-07 | 310 | 39 |
| Östra | 20-02-12 | 22-03-02 | 150 | 27 |
| Skövde | 21-05-03 | 22-03-04 | 123 | 16 |
| Linköping | 21-04-12 | 22-03-24 | 92 | 11 |
| Kalix | 21-02-25 | 22-02-14 | 63 | 6 |
| Piteå | 21-03-05 | 22-02-08 | 83 | 21 |
| Norrköping | 21-04-09 | 21-12-08 | 94 | 0 |
| Östersund | 21-02-03 | 22-02-28 | 93 | 30 |
| Oskarshamn | 21-03-11 | 21-09-08 | 20 | 0 |
| Lidköping | 21-05-04 | 21-07-20 | 17 | 0 |
| Ljungby | 21-03-18 | 21-06-28 | 21 | 4 |
| Lund | 21-03-04 | 21-05-21 | 30 | 9 |
| Jönköping | 21-04-28 | 22-02-21 | 87 | 6 |
| Uppsala | 21-11-30 | 22-03-17 | 34 | 2 |
| Sunderbyn | 21-04-12 | 22-03-31 | 97 | 7 |
| Falun | 21-04-01 | 22-03-25 | 112 | 6 |
| Hudiksvall | 21-06-14 | 21-10-25 | 44 | 1 |
| Gävle | 21-05-20 | 22-03-28 | 74 | 3 |
| Värnamo | 21-04-29 | 22-02-18 | 20 | 1 |
| Sundsvall | 21-04-12 | 22-03-31 | 194 | 14 |
| Gällivare | 21-10-04 | 22-03-24 | 20 | 3 |
| S:t Göran | 21-09-13 | 22-05-12 | 133 | 11 |
| Kungälv | 21-10-04 | 21-10-28 | 8 | 0 |
| Kalmar | 21-10-18 | 22-03-30 | 43 | 6 |
| Visby | 21-09-27 | 22-03-21 | 39 | 6 |
| Hässleholm | 21-11-08 | 22-03-25 | 26 | 1 |
| Angered | 21-09-29 | 22-05-31 | 55 | 2 |
|  |  |  |  |  |
| Total |  |  | 2082 | 232 |

Cardiac rehabilitation centres with patients screened = 27. Cardiac rehabilitation centres with patients included = 23.
